# Comparative Pathogenesis of Three Human and Zoonotic SARS-CoV Strains in Cynomolgus Macaques

**DOI:** 10.1371/journal.pone.0018558

**Published:** 2011-04-20

**Authors:** Barry Rockx, Friederike Feldmann, Douglas Brining, Don Gardner, Rachel LaCasse, Lisa Kercher, Dan Long, Rebecca Rosenke, Kimmo Virtaneva, Daniel E. Sturdevant, Stephen F. Porcella, John Mattoon, Michael Parnell, Ralph S. Baric, Heinz Feldmann

**Affiliations:** 1 Laboratory of Virology, Division of Intramural Research, National Institute of Allergy and Infectious Diseases, National Institutes of Health, Hamilton, Montana, United States of America; 2 Rocky Mountain Veterinary Branch, Division of Intramural Research, National Institute of Allergy and Infectious Diseases, National Institutes of Health, Hamilton, Montana, United States of America; 3 Genomics Unit, Research Technologies Section, Division of Intramural Research, National Institute of Allergy and Infectious Diseases, National Institutes of Health, Hamilton, Montana, United States of America; 4 Department of Veterinary Clinical Sciences, Washington State University, Pullman, Washington, United States of America; 5 Department of Epidemiology, University of North Carolina, Chapel Hill, North Carolina, United States of America; 6 Departments of Pathology and Microbiology and Immunology, University of Texas Medical Branch, Galveston, Texas, United States of America; University of Georgia, United States of America

## Abstract

The severe acute respiratory syndrome (SARS) epidemic was characterized by increased pathogenicity in the elderly due to an early exacerbated innate host response. SARS-CoV is a zoonotic pathogen that entered the human population through an intermediate host like the palm civet. To prevent future introductions of zoonotic SARS-CoV strains and subsequent transmission into the human population, heterologous disease models are needed to test the efficacy of vaccines and therapeutics against both late human and zoonotic isolates. Here we show that both human and zoonotic SARS-CoV strains can infect cynomolgus macaques and resulted in radiological as well as histopathological changes similar to those seen in mild human cases. Viral replication was higher in animals infected with a late human phase isolate compared to a zoonotic isolate. While there were significant differences in the number of host genes differentially regulated during the host responses between the three SARS-CoV strains, the top pathways and functions were similar and only apparent early during infection with the majority of genes associated with interferon signaling pathways. This study characterizes critical disease models in the evaluation and licensure of therapeutic strategies against SARS-CoV for human use.

## Introduction

Severe acute respiratory syndrome coronavirus (SARS-CoV) emerged in 2002 and 2003, resulting in about 8,000 human infections with an 11% case fatality rate [1]. SARS-CoV is a zoonotic pathogen that originated from bats and either entered the human population directly and/or cycled through palm civets and raccoon dogs as intermediate hosts [2], [3]. Advanced age was significantly associated with increased SARS-related pathogenicity and deaths due to rapidly progressive respiratory compromise (acute respiratory distress syndrome [ARDS]) [1], [4].

Based on epidemiological studies the SARS-CoV epidemic was characterized by different phases: zoonotic, early, middle and late phases [2]. The zoonotic phase was characterized by strains isolated from palm civets and raccoon dogs in live animal markets. The early phase was characterized by several independent human cases, likely due to zoonotic transmission. The middle phase was characterized by initial human-to-human transmission, whereas the late phase was characterized by efficient human-to-human transmission and extensive global spread to over 30 countries.

The SARS-CoV spike (S) glycoprotein has been identified as the main mediator of virus entry and host range by binding to its receptor angiotensin 1-converting enzyme 2 (ACE-2) [5], [6]. A high mutation rate of the S glycoprotein was observed between the different isolates from both animals and humans and several amino acid changes have been identified as being critical for the transition from animal-to-human to human-to-human transmission [6], [7], [8].

The S glycoprotein has also been identified as a major target of protective immunity and as such has been the main focus of vaccine development [9], [10]. While most vaccine candidates have been developed using nearly identical isolates from the late phase in the epidemic, it is not clear whether these late phase isolates will provide robust protection against infection with zoonotic and early human phase isolates, the most likely source of future outbreaks. Therefore a heterologous challenge model is needed to test cross-protection efficacy of vaccine candidates.

Currently, several animal models for SARS-CoV exist, including mice, hamsters, ferrets and non-human primates [11], [12], [13], [14], [15], [16], [17]. While these models have been used for pathogenesis studies as well as vaccine development, the majority of these studies focused on isolates from the late phase of the outbreak. In fact, heterologous challenge of mice after vaccination against a late phase S glycoprotein offered only partial protection [18].

We previously characterized the in vitro and in vivo virulence of an isogenic panel of recombinant SARS-CoV isolates bearing the S glycoprotein from zoonotic, early, middle and late phase isolates [19]. The late and early human phase viruses replicated to high titers in human ciliated airway epithelial cultures whereas the zoonotic isolates did not. Interestingly, the early human (GZ02) and zoonotic phase (HC/SZ/61/03) isolates were lethal in an aged mouse model resulting in severe lung infection progressing to an early organizing phase of ARDS and death [19], [20].

In this study we compare the pathogenesis of three recombinant SARS-CoV isolates bearing the S glycoprotein of zoonotic, early and late phase isolates in cynomolgus macaques. The Urbani strain and recombinant Urbani SARS-CoV from our molecular clone have previously been tested in a non-human primate model [15] and served as a control in the current study. Clinical, virological and histological parameters as well as the host responses were compared to more fully understand the effect of S glycoprotein variation on virus replication and the host response to SARS-CoV infection with the ultimate goal of characterizing heterologous challenge models for SARS-CoV vaccine development. Importantly, all viruses replicated to near similar titers and induced similar pathologic changes of mild disease, demonstrating the availability of heterologous challenge viruses for future vaccine studies.

## Results

### Clinical, laboratory, and radiographical observations

Groups of 9 cynomolgus macaques were challenged via the intratracheal and intranasal routes with the recombinant Urbani, GZ02 and HC/SZ/61/03 SARS-CoV strains. No overt clinical symptoms were seen in any of the infected animals. In addition, no fever was detected on clinical exam days 1, 2, 4, 7, 10 and 14 post infection. Minor transient lymphopenia was seen on days 1 and 2 p.i. in all infected animals (data not shown). No significant changes were observed in blood chemistry.

**Figure 1:**
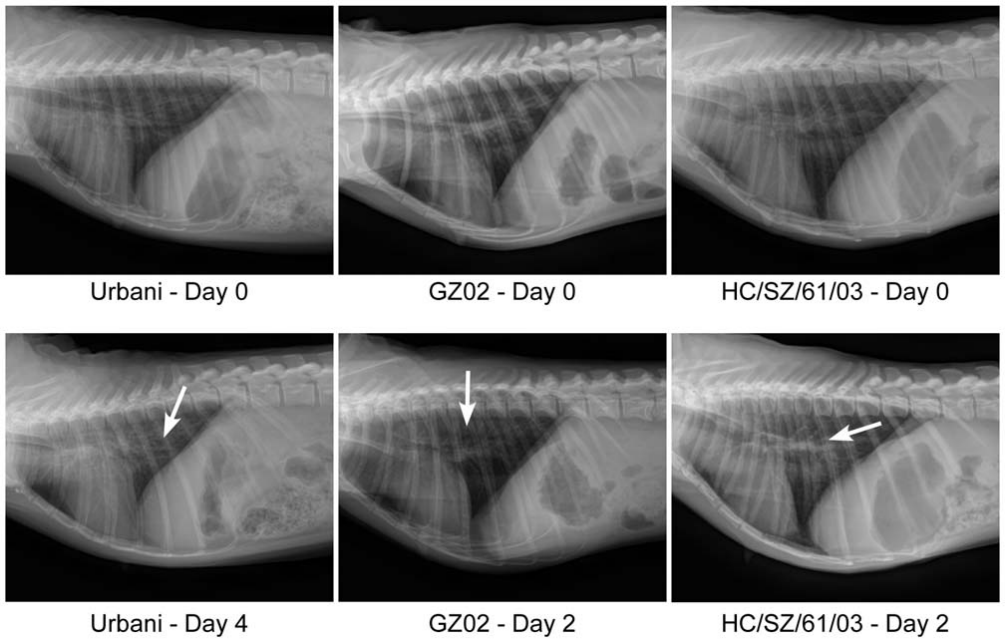
Radiological changes in SARS-CoV infected cynomolgus macaques. Radiologic progression of respiratory disease in Urbani, GZ02 or HC/SZ/61/03 infected animals during clinical exams at days 0, 2 or 4 post infection. White arrows represent infiltration and consolidation.

Respiratory disease development was also analyzed by radiographic imaging (X-ray) with first signs of mild interstitial pulmonary infiltration and peribronchial markings in the lungs of some animals infected with Urbani as early as day 1 p.i. (data not shown). On days 2 and 4 p.i. radiological changes in Urbani infected animals were very similar to day 1 with an additional decrease in conspicuity of the caudal vena cava (CVC; Fig. 1). These changes lasted up to day 11 in at least 1 of the animals. Radiological changes in HC/SZ/61/03 infected animals were very similar to Urbani infected animals. Interestingly, one animal showed a clear progression on day 2 with small ventral-most consolidation in left middle/caudal lung (right lateral view) and heavy peribronchial markings (Fig. 1). Notable improvement was seen by day 4 with mild interstitial infiltrates centrally, and unsharp CVC margins on both lateral views. Surprisingly, infection with GZ02 did not result in significant radiological changes early in infection, although mild increase in interstitial infiltrates could be observed in 1 animal on day 2 (Fig. 1).

Gross pathological findings during necropsies on days 1, 4 and 14 p.i. included enlarged cervical and bronchial lymph nodes, splenomegaly and adherence of lung lobes to pleura. No lesions were noticeable on the lungs of any of these animals.

### Virus replication

**Figure 2:**
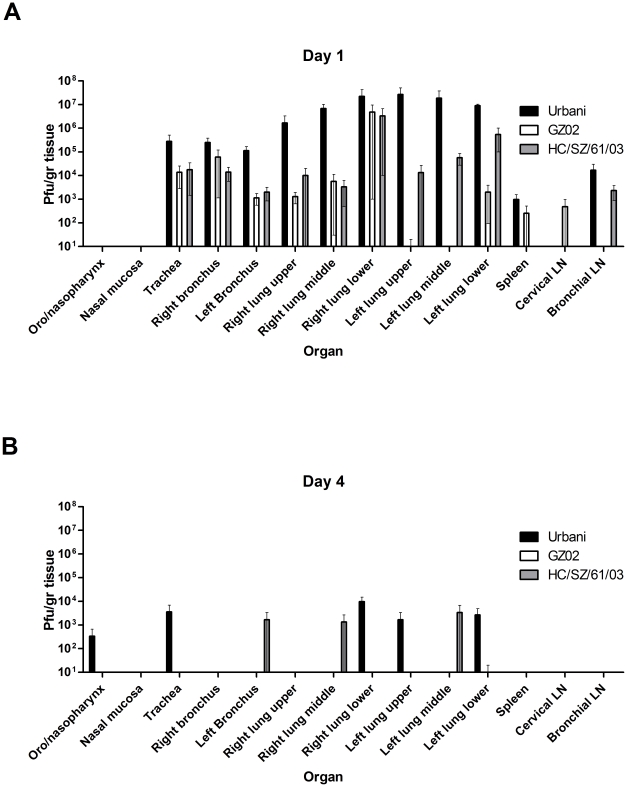
Virus replication by organ in SARS-CoV infected animals. Virus replication was determined in tissues on day 1 (A) and day 4 (B) by virus titration. Samples from three animals were assayed and analyzed and the mean titers were calculated as pfu/gr. The error bars represent the standard error of the mean.

Virus replication of all three SARS-CoV strains was mainly restricted to the respiratory tract (trachea, bronchi and lung lobes) and low levels of replication in spleen, cervical and bronchial lymph nodes (Fig. 2A and B). Viral replication peaked in the respiratory tract on day 1 p.i. (Fig. 2A) with a reduction in virus titers and numbers of virus positive tissues on day 4 p.i. (Fig. 2B), similar to our findings in mice [19]. No infectious virus could be isolated on 14 days p.i. in any of the tissues (Data not shown). Virus titers in Urbani infected animals were generally 1 to 2 logs higher compared to GZ02 and HC/SZ/61/03 infected animals. Viral RNA could be detected in the lungs of infected animals up to 14 days p.i. (data not shown). The levels of viral RNA were similar for each SARS-CoV strain at each time point but decreased over time as observed with the viral titers.

**Figure 3:**
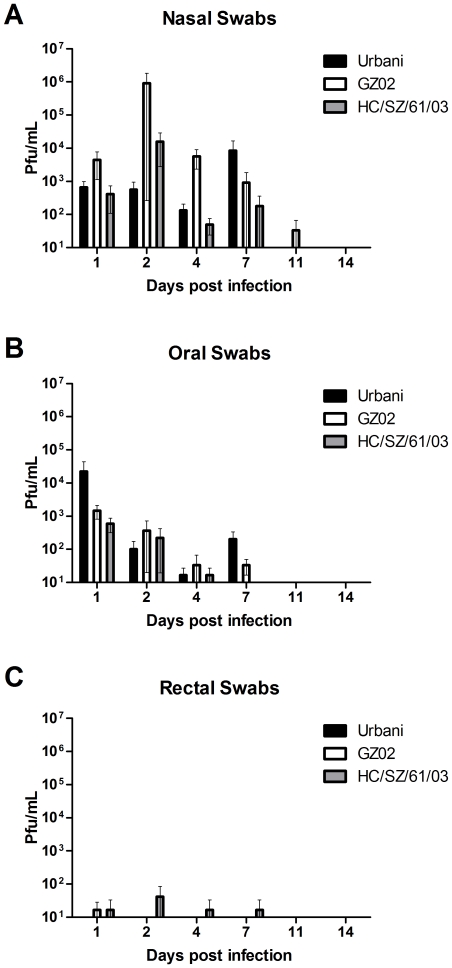
Virus shedding in SARS-CoV infected animals. Virus replication was determined in nasal (A), oral (B) and rectal (C) swabs of SARS-CoV infected animals by virus titration. Samples from three animals were assayed and the mean titers were calculated as pfu/mL. The error bars represent the standard error of the mean.

As a measure of virus shedding, nasal, oral and rectal swabs were assayed for infectious virus. In nasal swabs, virus titers peaked by day 2 but could be detected up to day 7 and 11 p.i. (Fig. 3A) Virus titers in oral swabs peaked on day 1 and were on or below detectable titers by day 4 p.i. (Fig. 3B). Low levels of virus replication could be observed in rectal swabs but only in a few animals (Fig. 3C).

### Histopathology and Immunohistochemistry

**Figure 4:**
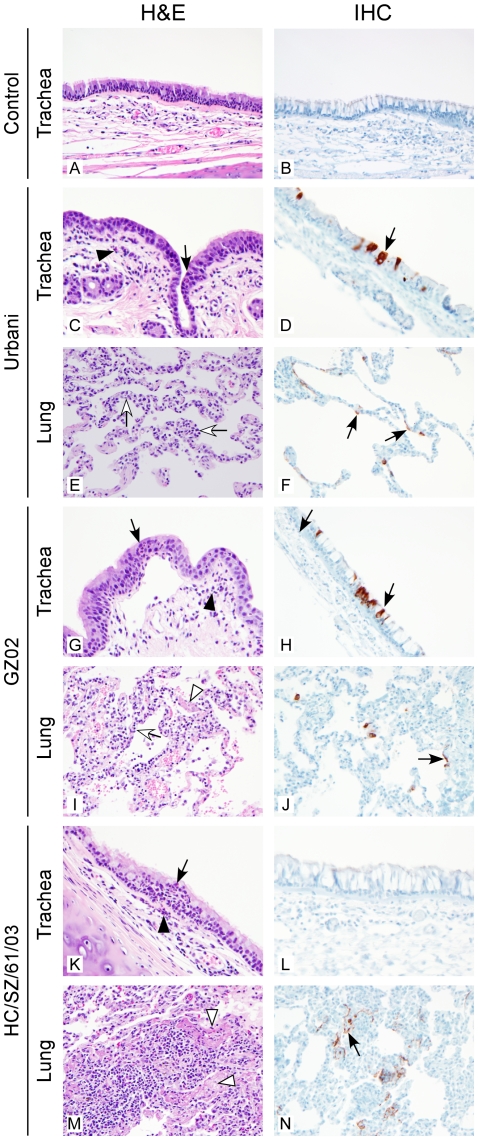
Histological changes and virus tropism in the respiratory tract of SARS-CoV infected animals on day 1. Controls (A, B), Urbani (C, D, E, F), GZ02 (G, H, I, J) and HC/SZ/61/03 (K, L, M, N) infected animals were euthanized on day 1 and trachea (A, B, C, D, G, H, K, L) and lung (E, F, I, J, M, N) sections were stained with H&E (A, C, E, G, I, K, M) or immunohistochemical (IHC) detection of SARS-CoV nucleoprotein (B, D, F, H, J, L, N) as described in Materials and Methods. H&E stained sections of trachea (A, C, G, K) with minimally increased numbers of lymphocytes, macrophages and neutrophils within the tracheal submucosa (black arrowhead) and occasional extension of neutrophils into epithelium (black arrow). IHC stained sections of trachea reveal occasional positively stained epithelial cells in Urbani (D) and GZ02 (H) infected animals; no positive IHC staining was detected in trachea sections from control (B) or HC/SZ/61/03 (L) infected animals; H&E stained lung sections (E, I, M) with alveolar septal walls expanded by lymphocytes, macrophages and few neutrophils (open arrow, E, I) and within alveoli (K); (I, M; open arrowhead = fibrin). IHC staining revealed occasionally positively stained cells with alveolar pneumocyte morphology (F, J, N). Original magnification = 400×; panels B, E and J are increased 133%.

**Figure 5:**
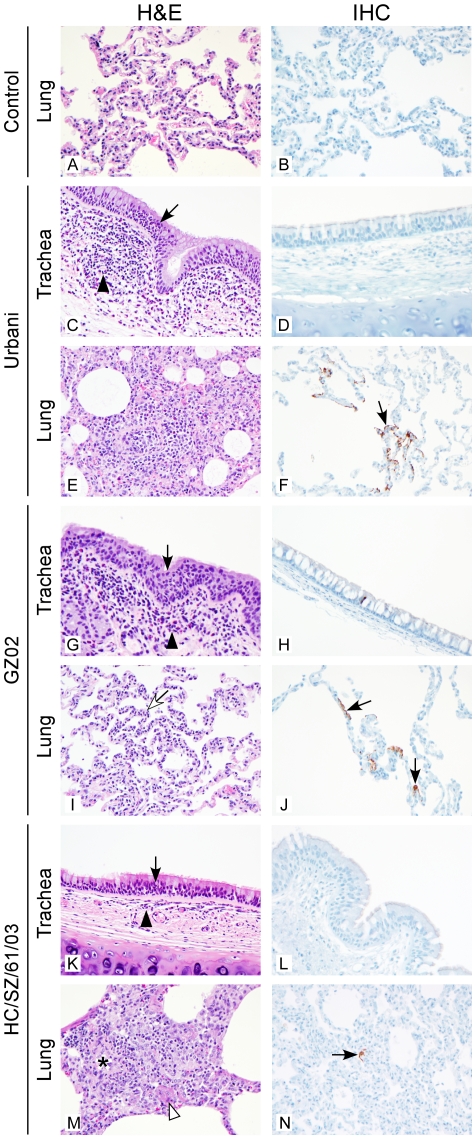
Histological changes and virus tropism in the respiratory tract of SARS-CoV infected animals on day 4. Controls (A, B), Urbani (C, D, E, F), GZ02 (G, H, I, J) and HC/SZ/61/03 (K, L, M, N) infected animals were euthanized on day 4 and trachea (C, D, G, H, K, L) and lung (A, B, E, F, I, J, M, N) sections were stained with H&E (A, C, E, G, I, K, M) or immunohistochemical (IHC) detection of SARS-CoV nucleoprotein (B, D, F, H, J, L, N) as described in Materials and Methods. H&E stained sections of trachea (C, G, K) with lymphocytes, macrophages, eosinophils and neutrophils within submucosa (black arrowhead) and intraepithelial neutrophils (black arrow). IHC stained sections of trachea (D, H, L) with no positive staining of tracheal epithelium. H&E stained sections of lung (A, E, I, M) with alveolar and perivascular aggregates of macrophages and lymphocytes (E, I, M) and same cells expanding alveolar septae (I, open arrow). (M, asterisk = blood vessel; open arrowhead = fibrin). IHC stained sections of lung (F, J, N) with occasional positively stained pneumocytes (black arrow). Original magnification = 400×; panels C, G and K are increased 133%.

Animals infected with any of the three strains of SARS-CoV showed mild inflammation within the submucosa, and occasional extension into the mucosa of the trachea and bronchi at day 1 p.i. compared to controls (Fig. 4A, C, E, G, I, K, M and Fig. 5A). Mild perivascular inflammation was observed within the lungs as well as a multifocal, minimal to mild increase of lymphocytes, macrophages and fewer neutrophils and eosinophils within the alveolar septae and alveoli (Fig. 4E, I and M). Rarely, alveolar fibrin, edema and minimal hemorrhage were observed. Urbani viral protein could be detected in tracheal epithelial cells and in alveolar pneumocytes at day 1 p.i. (Fig. 4D and F). Animals infected with either the GZ02 or HC/SZ/61/03 strains showed multifocal, mild to moderate (severity increased compared to Urbani strain lesions), subacute and eosinophilic pneumonitis, alveolitis, and occasional bronchitis and bronchiolitis at day 1 p.i. (Fig. 4I and M). In some animals, perivasculitis with edema was observed along with occasional alveolar fibrin deposits and type II pneumocyte hyperplasia. GZ02 viral protein could be detected in tracheal epithelial cells and in low numbers of macrophages and alveolar pneumocytes in the lung (Fig. 4H and J). HC/SZ/61/03 viral protein was not detected in the trachea (Fig. 4L), despite the presence of infectious virus by titration (Fig. 1A), but could be detected in alveolar pneumocytes in the lung at day 1 p.i. (Fig. 4N).

By day 4 p.i., a persistence of perivasculitis, peribronchiolitis and peribronchitis was observed with decreased incidence of alveolar septal pneumonitis and alveolitis in animals infected with all 3 SARS-CoV strains and inflammatory cells consisted mostly of macrophages and lymphocytes with few neutrophils (Fig. 5A, C, E, G, I, K and M). In animals infected with HC/SZ/61/03, occasional mild accumulations of fibrin and edema within the alveoli could be observed (Fig. 5M). No virus positive cells were observed in the trachea at 4 days p.i. in any of the animals (Fig. 5D, H and L); however, decreased numbers of alveolar pneumocytes were still positive for viral protein in the lungs of SARS–CoV infected animals compared to day 1 (Fig. 5F, J and N).

### Host gene expression

**Figure 6:**
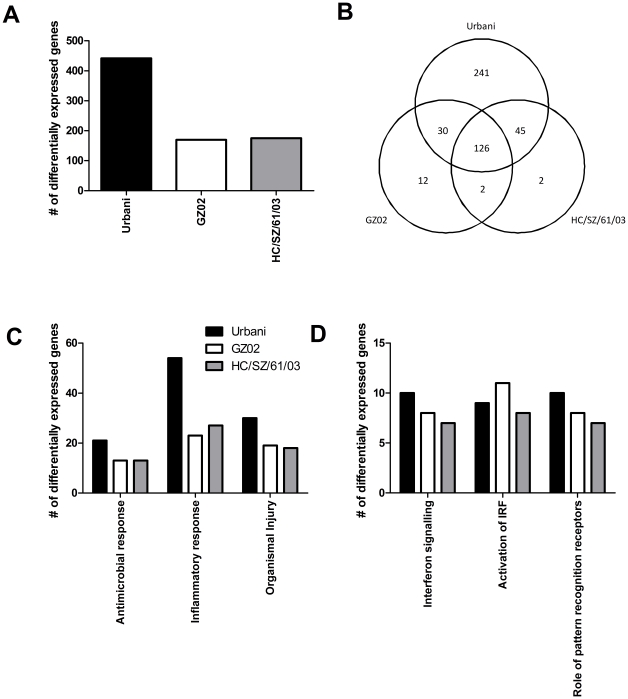
Host gene expression on day 1 after SARS-CoV infection. (A) The number of differentially expressed genes are the result of a comparison of gene expression in the lungs of SARS-CoV infected cynomolgus macaques versus gene expression in the lungs of mock-infected animals, and genes were included if they met the criteria of an absolute change of ≥2-fold (P≤0.05. (B) The Venn diagram shows the overlap of unique and common genes with ≥2-fold (P≤0.01) between Urbani, GZ02 and HC/SZ/61/03. Functional annotations (C) and canonical pathways (D) were determined in order to better understand the biological functions associated with SARS-CoV infection in cynomolgus macaques.

We previously showed that the host response was different between mice infected with the 3 different spike glycoprotein variants [20]. While clinically no differences were observed between the 3 viruses used in this study, virus replication was different (Fig. 2) suggesting that there may be differences in host responses. When comparing the host gene expression in lung tissue of the 3 virus infected groups with the host response of control animals, differentially expressed genes were only observed on day 1 p.i. with the highest number of differentially expressed genes in Urbani infected animals (Fig. 6A). At 1 day p.i. the majority of differentially expressed genes associated with GZ02 and HC/SZ/61/03 infection were common for all three viruses (Fig. 6B). Whereas the majority of genes associated with Urbani infection were unique.

In order to better understand the biological functions associated with the host response to SARS-CoV infection, the functional annotations were determined for the significant differentially expressed genes using IPA. Interestingly, although Urbani infection resulted in a higher number of differentially expressed genes compared to GZ02 and HC/SZ/61/03 infected animals, both the top 3 functions and canonical pathways were identical for all 3 SARS-CoV strains. The top functions included antimicrobial response, inflammatory response and organismal injury (Fig. 6C), whereas the top 3 canonical pathways included the interferon signaling, IRF activation and pattern recognition receptor pathways (Fig. 6D). Fold changes in genes involved in these pathways were generally higher in Urbani infected animals compared to GZ02 and HC/SZ/61/03 infected animals (Table 1) and included interferon inducible genes like interferon-induced protein with tetratricopeptide repeats (IFIT) 1 and 3, myxovirus resistance 1 (MX1) and others, as well as signal transducers and activators of transcription (STATs) and some of the highest differentially expressed genes included CXCL10, CXCL11, ISG15 and MX1.

**Table 1:** The top differentially upregulated genes belonging to the interferon signaling and immune response pathways.

			Fold Change		
	Gene Symbol	RefSeq Transcript ID	Urbani	GZ02	HC/SZ/61/03
Interferon signalling	IFIT1	XM_001086535	18	14	14.6
	IFIT3	XM_001086192	24.9	16.6	19.6
	IFITM1	XM_001085444	7.8	6.5	6.7
	IRF1	XM_001104294	8.8	6.9	-
	ISG15	XM_001088541	57.4	51	45.4
	MX1	XM_001107646	46	39.4	21.9
	OAS1	XM_001110682	23	11.5	14.4
	SOCS1	XM_001104595	5.5	-	-
	STAT1	XR_009921	6.2	6	6.4
	STAT2	XM_001115072	8.6	7.1	5.1
	TAP1	XM_001115506	3.7	-	-
Immune response	CCL8	NM_001032851	20.3	8.6	14
	CXCL10	NM_001032892	35.6	22.4	14.7
	CXCL11	NM_001032950	15.9	25.2	22
	IL-1ra	XM_001091833	6.9	11	6.7
	IL-29	XM_001085768	7.5	2.9	3.5

### Cytokine production

**Figure 7:**
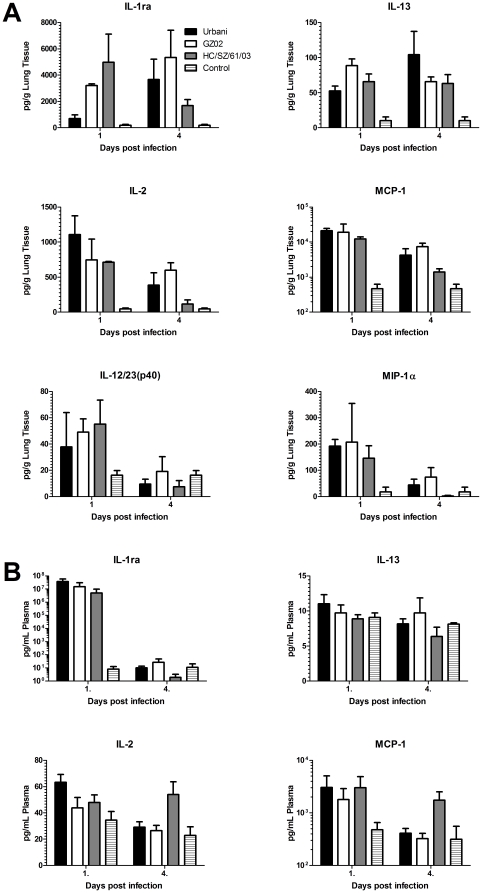
Cytokine levels in lung and blood of SARS-CoV infected cynomolgus macaques. The concentrations of cytokines were determined in lung homogenates (A) and plasma (B) on days 1 and 4 post infection as described in the materials and methods. Concentrations are expressed as picogram (pg) cytokine per gram lung tissue or mL plasma. The error bars represent the standard error of the mean.

In order to confirm mRNA expression kinetics of cytokines at the translational level, the protein levels of pro and anti-inflammatory cytokines were determined in the lung homogenates and plasma of control and SARS-CoV infected animals. While mRNA expression of IL-1ra, MCP-1 and Mip-1a was increased in SARS-CoV infected animals, this was not significant and therefore not included in the analyses described above. However in agreement with the overall host gene expression kinetics, the levels of IL-1ra, IL-2, IL12/23 (p40), IL-13, MCP-1 and Mip-1α in lung tissue were elevated in animals infected with the different SARS-CoV strains compared to those in control animals on days 1 or 4 p.i. peaking on day 1 p.i. (Fig. 7A) but not on day 14 p.i. (data not shown). Cytokine levels in blood generally did not correlate with those found in the lung with the exception of MCP-1 which peaked on day 1 p.i. (Fig. 7B). No significant differences were found between the 3 SARS-CoV strains.

## Discussion

SARS-CoV is a zoonotic pathogen that can cause severe respiratory disease in humans [1]. The evolution of the virus throughout the epidemic was characterized by a high mutation rate in the S glycoprotein and this adaptation correlated with increased pathogenicity in humans. We previously showed that mutations in the S glycoprotein can result in differences in virulence in an aged mouse model of SARS-CoV infection [19], [20]. These mutations also differentially affected replication in primary human airway epithelial cultures showing that in contrast to zoonotic SARS-CoV strains, human strains replicated efficiently in these cultures [19]. In this study we used a panel of these recombinant SARS-CoV bearing variant S glycoproteins to develop new models of SARS-CoV infection in non-human primates that could be used to test vaccine candidates and therapeutics against heterologous challenge.

Several groups have shown that cynomolgus macaques can be infected with SARS-CoV, although the clinical presentation is variable [15], [21], [22], [23]. However, these initial studies did not control for the age of the animals. Advanced age significantly increases the severity of SARS-CoV infection and was correlated with increased morbidity and mortality in humans [1]. In a recent study, SARS-CoV infection in 10–19 year old cynomolgus macaques resulted in decreased activity and mildly labored breathing in some animals [24]. In addition, pulmonary consolidation was observed as well as diffuse alveolar damage. In this study we infected 8–15 year old cynomolgus macaques with 3 recombinant SARS-CoV bearing S glycoprotein variants however the increased age did not result in overt clinical symptoms in our cynomolgus macaque model. In comparison, we previously infected cynomolgus macaques with the newly emerged H1N1 influenza virus which resulted in increased respiratory rates, nasal discharge, mild-moderate radiographical changes and gross pathologic lesions in the lungs [25]. One possible explanation for the differences observed between the 2 SARS-CoV studies is the use of different SARS-CoV strains. In the current study we used the Urbani strain, whereas Smits *et al* used a different strain of SARS-CoV (HK-39), isolated from one of the earliest Hong Kong patients, which contains several mutations throughout the genome [26]. These observed a.a. changes between the Urbani and HK-39 strain may affect the pathogenesis in the cynomolgus macaque model. In this study the recombinant SARS-CoV strains bearing the variant S glycoproteins were all constructed in the Urbani background. We had previously hypothesized that the 3 recombinant SARS-CoV strains would show differences in replication in non-human primates due to the fact that both the Urbani and GZ02 strains replicated efficiently in human airway epithelium, whereas the zoonotic strains including HC/SZ/61/03 did not [19]. This was in agreement with observations in human cases of SARS-CoV infection where infection with late phase SARS-CoV strains resulted in more severe disease and efficient transmission compared to the mild sporadic cases during the zoonotic phase of the epidemic [2]. It has been shown that the S glycoproteins of these strains have adapted to more efficient binding to its receptor ACE2 [7], [8], [27]. Indeed the epidemic Urbani strain replicated to higher titers compared to GZ02 and HC/SZ/61/03, and shedding of these viruses could be detected primarily in the nasal and oral swabs as a measure for potential transmission. However, unlike infection in mice [20], no differences were found in the cell tropism with all 3 SARS-CoV strains targeting primarily type II pneumocytes. While no viral antigen was detected in the trachea of HC/SZ/61/03 infected animals, the recovery of infectious virus from this tissue suggests that the absence of antigen is unlikely to be due to a difference in tropism but rather due to limitations in detection.

In addition, the SARS-CoV S glycoprotein has been implicated in the downregulation of ACE2 expression [20], [28], [29]. This downregulation can cause a deregulation of the renin-angiotensin system and an exaggeration of acute lung failure [30]. We hypothesize that the SARS-CoV S glycoproteins from the different zoonotic and human phases of the epidemic may differentially down-regulate ACE2, resulting in the observed disconnect between virus replication and pathogenesis. However unlike in the mouse model, no differences in ACE2 expression could be observed by gene expression or immunohistochemistry (data not shown) in cynomolgus macaques, possibly due to the mild disease progression observed in the current model. While the S glycoprotein is a major virulence factor [8], [19], [27], it cannot be excluded that the additional mutations in the rest of the genomes of the GZ02 and HC/SZ/61/03 strains could be necessary for a more virulent phenotype in the cynomolgus macaque model.

In the current model, no gross pathological lesions were evident in the lungs of these animals. Interestingly, SARS-CoV infection did result in radiological changes including pulmonary infiltration and peribronchial markings early during infection. Although observed earlier, it is similar to what was seen in mild human cases [31] as well as in animals infected by the IN route [15]. In contrast, animals infected by the IN and intrabronchial (IB) route showed early radiological changes in the lower lung lobes [15]. One explanation for the early detection of radiological changes in the current model is that animals were infected intratracheally with a high dose of SARS-CoV. Intratracheal instillation resulted in the presence of a high virus load in the trachea and bronchi of the animal, and subsequent peribronchial markings observed early on during infection. Natural infection in human cases would result from exposure to a lower virus dose and a less efficient route [32], [33]. In contrast, infection by the intrabronchial route results in initial virus replication in the interstitium, resulting in radiological changes in the lower lobes early during infection [15]. The radiological changes correlated with the observed histopathology in the lungs. While this model did not exhibit the severe diffuse alveolar damage (DAD), edema and hyaline membrane formation associated with severe human cases of SARS-CoV infection [34], alveolitis with edema, fibrin deposits and type II pneumocyte hyperplasia was observed in some animals infected with the GZ02 and HC/SZ/61/03 strains but not in Urbani infected animals. This is in agreement with observations in mice where increased severity of lung pathology also did not correlate with virus replication in the lung and the Urbani strain replicated to 1–2 log higher titers compared to the GZ02 and HC/SZ/61/03 strains.

In human cases, upregulation of IFN pathways plus robust antiviral IFN-stimulated gene (ISG) expression was observed early during SARS-CoV infection [35]. In addition, several animal models have shown that an acute and exacerbated host response results in acute lung injury [20], [24]. In the current study we observed an early upregulation of genes associated with IFN signaling and activation of IFN related pathways. This early upregulation has previously been shown in mouse and non-human primate models of SARS-CoV infection as well as influenza infection [20], [24], [36], [37]. In particular, early enhanced expression of interferon-inducible protein-10 (CXCL-10) and monocyte chemoattractant protein-1 (MCP-1) have been correlated with an adverse outcome during SARS-CoV infection in humans [38], [39], which was also highly upregulated in this study. Unfortunately, due to the animal-to-animal variation, differentially expressed genes could only be identified on day 1 p.i. Interestingly, no differences were observed in the pathways involved in all 3 SARS-CoV strains, although the number of genes involved in these pathways were higher in Urbani infected animals compared to GZ02 or HC/SZ/61/03 infected animals. In addition, the fold-changes were also greater following infection with the Urbani strain compared to GZ02 and HC/SZ/61/03. The higher upregulation of host genes following Urbani infection correlates with the observed replication. In agreement with the observed host gene response, expression of both inflammatory and anti-inflammatory mediators such as IL-1ra, IL-12, IL-13 and MCP-1 was upregulated in the lungs of infected animals on day 1 p.i. Upregulation of these genes was previously observed in serum and peripheral blood mononuclear cells in human cases of SARS [40], [41], [42]. In addition, MCP-1 was also found to be upregulated in plasma from H1N1 influenza infected macaques [25].

In conclusion, this study demonstrates that cynomolgus macaques can be infected with zoonotic and early human epidemic phase SARS-CoV strains, as shown by the presence of infectious virus in the respiratory tract at days 1 and 4. In addition we show that infection results in prolonged shedding of the virus in nasal and oral secretions up to 7 days post challenge as a potential method of transmission. Finally, infection with the 3 SARS-CoV strains induces host responses early during infection. These data show that the new cynomolgus macaque models will be important in testing the efficacy of vaccine candidates or therapeutics against antigenically distinct SARS-CoV strains [43]. To date, the majority of vaccine studies have focused on the epidemic Urbani strain which provide limited protection against heterologous strains, especially in senescent populations that are more at risk for severe disease [10], [18]. The epidemic SARS-CoV strains are extinct; however zoonotic strains may still emerge from animal reservoirs like bats, palm-civets and raccoondogs. Therefore a SARS-CoV vaccine should confer long-term protection against replication and shedding of both the epidemic strains and any zoonotic strain that may emerge.

## Materials and Methods

### Viruses and cells

The generation and characterization of each of the recombinant SARS-CoV bearing variant S glycoproteins (Urbani, GZ02 and HC/SZ/6103) has been described previously [19]. All viruses were propagated on Vero E6 cells in Dulbecco's minimal essential medium (DMEM; Invitrogen), supplemented with 10% fetal bovine serum, L-Glutamine, penicillin (10,000 IU/mL) and streptomycin (10,000 IU/mL) at 37°C in a humidified CO_2_ incubator. All work was performed in a Class II Biosafety Cabinet under BSL-3 conditions at RML, DIR, NIH.

### Animals and Ethical Statement

Healthy, adult female cynomolgus macaques (Macaca fascicularis) were handled in an ABSL-4 containment laboratory at RML, DIR, NIH. Research was conducted in compliance with the Animal Welfare Act and other federal statutes and regulations relating to animals and experiments involving animals, and adhered principles stated in the Guide for the Care and Use of Laboratory Animals, National Research Council, 1996. The facility where this research was conducted (RML) is fully accredited by the Association for the Assessment and Accreditation of Laboratory Animal Care International and has an approved OLAW Assurance #A4149-01. Research was conducted under a protocol approved by the Institutional Animal Care and Use Committee (IACUC) at RML. All steps were taken to ameliorate the welfare and to avoid the suffering of the animals in accordance with the “Weatherall report for the use of non-human primates” recommendations. Animals were housed in adjoining individual primate cages allowing social interactions, under controlled conditions of humidity, temperature and light (12-hour light/12-hour dark cycles). Food and water were available ad libitum. Animals were monitored (pre- and post-infection) and fed commercial monkey chow, treats and fruit twice daily by trained personnel. Environmental enrichment consisted of commercial toys. All procedures were conducted by trained personnel under the supervision of veterinarians and all invasive clinical procedures were performed while animals were anesthetized. Early endpoint criteria, as specified by IACUC approved score parameters, were used to determine when animals should be humanely euthanized.

### Animal procedures

Thirty female Cynomolgus macaques (*Macaca* fascicularis; ages 8–15) weighing between 2.5 and 4 kg were distributed evenly in regards to age and weight over four groups (9 animals per virus group and 3 animals in a control group). Animals were infected with either Urbani, GZ02 or HC/SZ/61/03 under anesthesia through a combination of intratracheal (4 ml) and intranasal (0.5 ml per nostril) installation with a suspension containing 2×10^6^ plaque forming units (pfu) per ml DMEM (total infectious dose = 1×10^7^ pfu) of each of the 3 different SARS-CoV strains. Three animals were mock infected with virus culture medium only (DMEM+10% FCS+p/s). Animals were anesthetized for challenge, clinical examination, temperature, respiration rate, chest radiographs, blood draw and swabs of nasal, oral and rectal mucosa on days 0, 1, 2, 4, 7, 11 and 14 p.i. Three animals from each infected group and 1 control animal were euthanized and necropsied on days 1, 4 and 14 p.i. with collection of clinical specimens from oral and nasal mucosa, trachea, left and right bronchi, left and right lung (upper, middle and lower lobes), spleen, liver, kidney, heart, cervical and bronchial lymph nodes.

### Chest radiographs

A mobile digital radiography unit with a flat panel digital detector (Sound Technologies tru/Dr) and a portable x-ray generator (Poskom model PXP-HF) was used to acquire chest radiographs. The system operates on veterinary specific software system (Vetpacs). Animals were positioned for ventrodorsal radiography by using a Lexan v-shaped thoracic positioner. Radiographic views acquired included ventrodorsal, right lateral, and left lateral thoracic images while under anesthesia. The digital images were interpreted and assigned a numeric score by a veterinary radiologist who was blinded to the study design (JM). A score on a scale of 0–3 based on severity of radiographic changes were assigned to each set of radiographs (ventrodorsal, right lateral and left lateral) at each time point. Radiographs were evaluated and scored using the following criteria: Grade 0, normal examination; Grade 1, mild interstitial pulmonary infiltrates; Grade 2, moderate interstitial infiltrates, this may include partial cardiac border effacement and small areas of pulmonary consolidation (alveolar patterns and air bronchograms); and Grade 3, pulmonary consolidation as the primary lung pathology, seen as a progression from Grade 2 lung pathology.

### Hematology and blood biochemistry

Total white blood cell (WBC) count, lymphocyte, platelet, reticulocyte and red blood cell counts, hemoglobin, hematocrit values, mean cell volume, mean corpuscular volume and mean corpuscular hemoglobin concentrations were determined from EDTA blood with the HemaVet 950FS+ laserbased hematology analyzer (Drew Scientific). Serum biochemistry was analyzed from heparin blood using the blood chemistry analyzer, iSTAT1 (Abbott Point of Care). Using the EC8+ Cartridge, urea nitrogen (BUN), glucose, chloride, sodium, potassium, hematocrit, hemoglobin, pH, PCO2, TCO2, base excess (BEecf), and anion gap values were determined; creatinine values were evaluated using the Crea cartridges.

### Cytokine analysis

Concentrations of G-CSF, GM-CSF, IFNγ, IL-1β, IL-1ra, IL-2, IL-4, IL-5, IL-6, IL-8, IL-10, IL-12/23 p40, IL-13, IL-15, IL-17, MCP-1, MIP-1α, MIP1β, sCD40L, TGFα, TNFα and VEGF in plasma of animals was determined on days 0, 1, 2, 4, 7, 11 and 14 p.i. and in lung homogenates on days 1, 4 and 14 p.i. using a Non-Human Primate Cytokine MILLIPLEX map kit (Millipore Corp.) as described by the manufacturer. Samples were read using a Bio-Plex 200 system (Bio-Rad).

### Virus titrations

Nasal, oral and rectal swabs were collected and stored in 1 ml DMEM and vortexed for 30 seconds. Tissue samples were weighed and homogenized in 10 equivalent volumes of DMEM to generate a 10% solution. The solution was centrifuged at 10,000 rpm under aerosol containment in a table top centrifuge for 5 min. Supernatants from swabs and tissue homogenates were serially diluted in DMEM, and 200 µl volumes of each dilution were placed onto monolayers of Vero-E6 cells in six-well plates. Following 1 hour of incubation at 37°C, the cells were overlaid with 0.8% agarose-containing medium. Two days later, the plates were stained with crystal violet and the plaques were counted. Titers were calculated as plaque forming unit (pfu) per ml or gr for swabs and tissues respectively. Titers were graphed using GraphPad Prism.

### Histopathology and immunohistochemistry

All tissues were inactivated and fixed in 10% phosphate-buffered formalin for at least 7 days prior to being processed by conventional methods, embedded in paraffin, sectioning at 5 µm thickness and hematoxylin and eosin staining. Slides were evaluated by a veterinary pathologist (D.G.). Tissues for immunohistochemistry were stained on the Discovery XT automated stainer (Ventana Medical Systems) using the anti-SARS-nucleoprotein antibody (Imgenex) and the DAB map detection kit (Ventana Medical Systems). Non-immune rabbit IgG was used as a negative staining control.

### Microarray analysis

Tissue samples were placed in RNAlater (Qiagen) for 11 days at 4°C. RNAlater was removed and samples were homogenized in 1 ml of Trizol for subsequent RNA extraction. Upon thawing, 200 µl chloroform was added, vials were vortexed, and centrifuged at 16,000× g for 15 min. RNA containing aqueous phase of 350–550 µl was collected from each sample and passed through Qiashredder column (Qiagen) at 21,000× g for 2 minutes to homogenize any remaining genomic DNA in the aqueous phase. RNA was purified using RNeasy 96 kit (Qiagen) as described previously except RNA samples were treated with DNase I during extraction [44].

An aliquot of sample RNA was used to quantify the three SARS-CoV strains (Urbani, GZ02, & HC/SZ/61/03). RNA extracted from a viral stock of the SARS-CoV Urbani strain was serial diluted and used as standard curve on all three Q-PCR plates. AgPath-ID One Step PCR kit (Ambion) was utilized with published SARS-CoV primers and probe [22]. A Q-PCR master mix was prepared according to manufacturers recommendations (Ambion). Viral gene expression was expressed as pfu equivalents per µg total RNA from lung tissues.

Five nanograms of RNA sample from tissue with comparable viral RNA levels within each group of animals was processed according to manufacturer's instructions using WT-Ovation™ Pico system RNA Amplification System (Nugen Inc.). Each sample was fragmented and labeled according to manufacturer's instructions for standard format anti-sense (AT) GeneChip arrays (Nugen Inc). Samples were hybridized onto Affymetrix GeneChip Rhesus Macaque Genome Arrays (Affymetrix) according to manufacturer's recommendations. Each chip was scanned using the Affymetrix 7Gplus GeneChip scanner to create the image files (dat). GeneChip Operating Software (GCOS v1.4) was then used to convert the image files to cell intensity data (cel files). The cel files were input into Partek Genomics Suite software (Partek, inc. St. Louis, Mo., v6.5 6091110) and quantile normalized to produce the significant probe set lists at the 0.05 significance level with multiple test corrections using the false discovery rate method to determine each probe set p-value. Functional annotations of significant differentially expressed genes were identified using Ingenuity Pathway Analysis (IPA; Ingenuity Systems). All microarray data is deposited in gene expression omnibus GSE23955 (http://www.ncbi.nlm.nih.gov/geo/) in accordance with proposed MIAME standards.
